# Raman Spectroscopy in the Diagnosis of Brain Gliomas: A Literature Review

**DOI:** 10.7759/cureus.79165

**Published:** 2025-02-17

**Authors:** Evgeny V Stupak, Vadim M Glotov, Arsen S Askandaryan, Sarah E Clancy, James C Hiana, Olga P Cherkasova, Vyacheslav V Stupak

**Affiliations:** 1 Department of Neurosurgery, Novosibirsk Research Institute of Traumatology and Orthopedics n.a. Ya.L. Tsivyan, Novosibirsk, RUS; 2 Department of Psychiatry, St. John's Episcopal Hospital, New York, USA; 3 College of Medicine, William Carey University College of Osteopathic Medicine, Hattiesburg, USA; 4 Department of Neurology, State University of New York Downstate Medical Center, New York, USA; 5 Laboratory of Terahertz Photonics, Institute of Automation and Electrometry, Siberian Branch of the Russian Academy of Sciences, Novosibirsk, RUS; 6 Automation and Computer Engineering Department, Novosibirsk State Technical University, Novosibirsk, RUS

**Keywords:** brain tumors cns tumors, cns neoplasm, cns tumor, glioblastoma, glioma, neuro-oncology, neurosurgery, raman spectroscopy, surface-enhanced raman spectroscopy, visible resonance raman spectroscopy

## Abstract

Raman spectroscopy (RS) is increasingly applied in medical fields to distinguish neoplastic from normal tissues, with recent advancements enabling its use in neurosurgery. This review explores RS as a diagnostic and surgical aid for brain gliomas, detailing its various modalities and applications. Through a comprehensive search in databases including PubMed, Google Scholar, and eLibrary, over 300 references were screened, resulting in 74 articles that met inclusion criteria. Key findings reveal RS’s potential in neuro-oncology for examining native biopsy specimens, frozen and paraffin-embedded tissues, and body fluids, as well as performing intraoperative assessments. RS offers promise for identifying gliomas, differentiating them from healthy brain tissue, and establishing precise tumor boundaries during resection.

## Introduction and background

Central nervous system (CNS) gliomas rank as the third leading cause of mortality in individuals aged 15 to 35 years and second in children under 15 years [1,2]. Glioblastomas, the most aggressive form of primary CNS tumors, are marked by rapid progression and poor prognosis, with median survival ranging from 8 to 20 months post-diagnosis despite intensive treatment [3]. This poor outlook stems largely from the infiltrative nature of malignant gliomas, including glioblastomas, which prevents complete surgical resection due to the lack of clear boundaries between tumor and healthy brain tissue. Current treatment for malignant brain tumors (MBTs) typically involves surgical resection followed by adjuvant radiation and chemotherapy [4,5].

The complexity of surgical removal and the high recurrence rates of glial tumors due to their infiltrative growth make surgical management challenging [6]. To address this, intraoperative technologies such as ultrasound and navigation systems are continually being enhanced, though each has specific limitations [7-10]. Fluorescent intraoperative navigation is commonly used; however, it relies on tumor tissue’s ability to accumulate fluorescent markers. In cases where this is ineffective, other diagnostic approaches are needed to accurately delineate tumor boundaries [6,11].

Despite advancements in intraoperative techniques to improve the extent of tumor resection, early detection of neoplasms and prevention of recurrence remain ongoing challenges [8,9,12]. Molecular genetic analysis has become essential for guiding clinical and surgical decisions and remains the gold standard for diagnosing brain neoplasms [13]. However, the high cost, labor intensity, and time requirements of molecular tests limit their use in intraoperative and rapid diagnostic settings. Consequently, a fast, automated method to accurately detect and differentiate tumors from healthy tissue is needed to assist pathologists in making precise diagnoses [14].

According to the WHO’s 2021 CNS tumor classification, alongside molecular genetic profiling of gliomas, the need for minimally invasive monitoring of tumor response to treatment has become increasingly critical, given the inadequacy of current prognostic markers for evaluating therapeutic outcomes [15-17].

Advancements in optical and molecular imaging have accelerated significantly in the past decade. Among these, optical spectroscopy stands out, offering insights into the intrinsic optical properties of tissues such as structural organization, nuclear density, fluorophore presence, and water content [18]. Light’s interaction with matter occasionally results in inelastic scattering, where photons gain or lose energy. This phenomenon, discovered by Indian scientist C.V. Raman in 1928, is known as Raman scattering or the Raman effect [19]. RS, a laser-based technique, leverages this effect to determine the molecular composition of tissue samples in seconds, enabling non-invasive molecular differentiation without dyes or extraction processes [20].

The Raman spectrum of cells and microorganisms is uniquely specific, often described as a “molecular fingerprint” useful for unambiguous identification. With in situ Raman imaging, specialized devices can transform these spectroscopic fingerprints into visual representations of molecular types [21].

One innovative RS-based application is stimulated Raman histology (SRH), which streamlines the acquisition and analysis of intraoperative histological data, aiding in swift surgical decision-making and potentially reducing operation times [22]. Utilizing a database of reference spectra for glial tumor components, this technique facilitates multidimensional tumor diagnosis and precise intraoperative localization [11].

## Review

**Figure 1:**
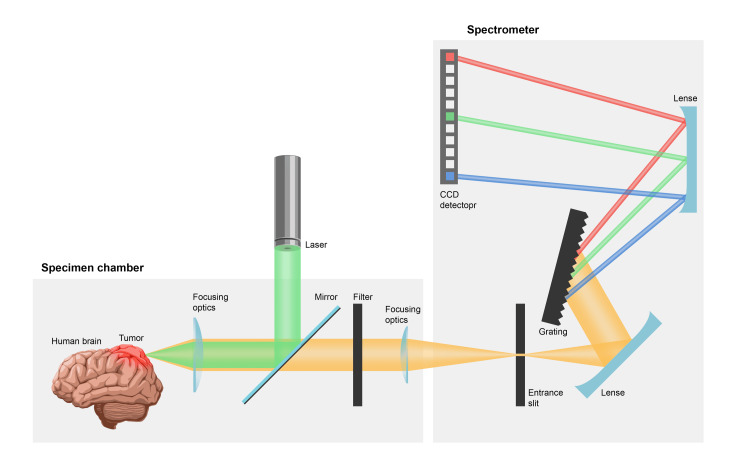
Schematic process of Raman spectroscopy Image Credits: Aleksey "Iva" Ivanchenko. Permission was obtained from the creator of the figure.

Raman spectroscopy (RS) has been used in biological and medical applications for over two decades. Technological advancements in RS instruments and statistical evaluation methods have facilitated the transition from ex vivo demonstrations to in vivo applications (Figure 1). For years, RS has proven effective in distinguishing neoplastic from normal tissues in patients with various cancers, including breast, stomach, cervical, oral, colorectal, and thyroid cancers [23-26]. Although RS’s clinical potential has been recognized for decades, its application in neurosurgery has only recently gained traction. With recent innovations, different forms of vibrational spectroscopy hold promise as breakthrough tools for neurosurgeons [27].

Types of Raman spectroscopy

In the last 15 years, 4 major literature reviews have highlighted the potential of various RS techniques in neurosurgery and biological research. One review identifies spontaneous Raman scattering and surface-enhanced Raman spectroscopy (SERS) as the most commonly applied imaging techniques. SERS, first identified by Fleischman M et al. in 1974 and later developed by Jeanmaire DL and Albrecht MG in 1977 [28-30], was comprehensively reviewed by Moskovits M in 2006 [31].

Two additional reviews focus on intraoperative handheld Raman probes, which are designed to maximize tumor resection accuracy. These reviews provide a detailed overview of current technologies, clinical trials, and future advancements needed to bring this technology into widespread clinical use. In vivo studies with Raman probes indicate RS’s ability to distinguish normal glial tissue from glioblastoma with high accuracy, sensitivity, and specificity. Additionally, nanosensors like gold nanoparticles have successfully enhanced RS’s capability to differentiate tumors from normal brain tissue using SERS [32].

The fourth review highlights RS’s potential to deliver label-free molecular information from tissues during surgery. Due to the complex microenvironment of brain tissue, data analysis remains a crucial factor for achieving high-performance Raman probe spectroscopy. The latest devices are now being tested in operating rooms, with their clinical application requiring interdisciplinary collaboration among physicians, engineers, and data scientists [33].

Applications of RS in neuro-oncology

A focused analysis of academic literature on Raman spectroscopy (RS) in neuro-oncology has identified several promising applications for diagnosing and surgically treating brain gliomas. These include the examination of native biopsy specimens, frozen tissue, paraffin-embedded tissue blocks containing brain gliomas, and body fluids such as blood plasma. RS also shows potential for rapid intraoperative testing, allowing differentiation between normal brain tissue and glial tumors. Additionally, RS can assist in defining the boundaries of gliomas during surgery to accurately distinguish tumor tissue from healthy brain tissue.

RS for the examination of native biopsy specimens, frozen tissue, paraffin-embedded tissue blocks, and body fluids

Several early studies explored RS for diagnosing brain gliomas, demonstrating its potential for high accuracy and rapid results. In 2010, Beljebbar et al. utilized RS for ex vivo and in vivo identification of normal versus tumor tissue in cases of glioblastoma, achieving high-quality spectral differentiation between the two. Using a portable Raman spectrometer with a microprobe and a signal acquisition time of five seconds, they achieved 100% accuracy, suggesting RS’s potential for early in vivo tumor diagnosis [33].

In 2013, Gajjar K et al. further examined RS’s ability to distinguish normal brain tissue from various tumor types-meningiomas, gliomas, and metastases through unique spectral “fingerprints” indicative of biochemical composition. This study used formalin-fixed, paraffin-embedded (FFPE) brain tissue blocks that were deparaffinized, mounted on slides, and dried for analysis with RS and Fourier-transform infrared attenuated total reflection (FTIR-ATR) spectroscopy. Both RS and IR methods successfully differentiated the histological structures and compositions of normal and tumorous tissues [34].

Bae K et al. (2018) developed an imaging technique using epi-detected hyperspectral stimulated Raman spectroscopy (SRS) based on spectral focusing for the label-free identification of glioblastoma biomolecular subtypes. This approach, requiring no preprocessing, generated hyperspectral Raman images at intervals of 7 cm-1 within 30 seconds, allowing detection of biochemical and morphological differences across glioblastoma types. Data analysis via principal component and linear discriminant analyses yielded diagnostic sensitivity and specificity rates of 96.7% and 88.9%, respectively [35].

In 2022, Colman H confirmed stimulated Raman histology (SRH) as a reliable method for intraoperative diagnosis, showing consistency with both frozen section analysis and histological examination, thus underscoring SRH’s potential as a viable intraoperative alternative [36].

Klamminger GG et al. (2021) examined SRH’s application on fresh and frozen tissues, finding it effective in these cases. However, they noted challenges with FFPE samples due to chemical processing, which can impede diagnostic accuracy [37].

A comparative study by Einstein EH et al. (2022) between SRH and frozen section histology demonstrated SRH’s effectiveness in 78% of analyzed samples, compared to 94% for traditional histology. Their results indicate that SRH’s diagnostic capabilities are non-inferior to those of frozen section analysis, supporting SRH as a promising tool for intraoperative diagnosis [38].

Di et al. (2021) conducted a blind, prospective cohort study involving 82 patients with post-brain tumor resection, with histological results diagnosing glioma in 21 cases. The study compared diagnostic accuracy and time-to-diagnosis between stimulated Raman histology (SRH) and traditional frozen section histology. The findings showed no significant difference in diagnostic accuracy between the methods (P = 1.00), but SRH significantly reduced diagnosis time (9.7 vs. 43 minutes, P < 0.0001), identifying key features for different glioma types [22]. In a 2020 study, Kowalska AA demonstrated that SERS could distinguish brain tumor samples from healthy tissue with 96% accuracy by analyzing three key spectral components. Significant spectral regions for this distinction included vibrations associated with L-tryptophan (1,450 and 1,278 cm-1), protein (1,300 cm-1), phenylalanine, and amide-I (1,005 and 1,654 cm-1) [39].

Hollon TC et al. (2020) conducted a multicenter prospective clinical trial using a convolutional neural network (CNN) trained on over 2.5 million SRH images. Their findings confirmed that SRH with CNN analysis was comparable to conventional histology, achieving an overall diagnostic accuracy of 94.6% versus 93.9%. This method enabled intraoperative brain tumor diagnosis within 150 seconds [40].

Livermore LJ et al. published several studies confirming RS's effectiveness in identifying brain gliomas in both fixed and unprocessed tissue samples. They also compared RS to 5-ALA-induced fluorescence for glioblastoma, finding RS superior, with RS providing a predictive accuracy of 0.24 (P = 0.0009), while 5-ALA-induced fluorescence had a sensitivity of 0.07, specificity of 1.00, and accuracy of 0.24 [13,41]. In a 2021 study, Sciortino T and colleagues performed spectral analysis on samples immediately after resection, distinguishing IDH-mutant from IDH-wildtype tumors with 87% accuracy. The Raman spectra showed specific lipid, collagen, DNA, and cholesterol/phospholipid signatures [42].

Galli R et al. (2019) applied RS to 209 tissue samples, using principal component analysis (PCA) for machine learning analysis to differentiate normal brain tissue from tumor tissue and to distinguish between glial tumor types. RS correctly identified normal brain tissue in 100% (7 of 7) and tumor tissue in 97% (195 of 202) of cases, achieving high diagnostic accuracy across primary glioblastoma (94%), recurrent glioblastoma (100%), astrocytoma (86%), and oligodendroglioma (90%) [43].

In 2022, Iturrioz-Rodríguez N investigated RS in the 1,000-1,300 cm-1 range to differentiate healthy astrocytes from glioma cells, achieving a mean accuracy of 92.5% with a PCA-linear discriminant analysis (LDA) model [44].

Kopec M et al. (2021) highlighted RS’s diagnostic potential across various brain tumor types, identifying universal biomarkers such as carotenoid bands (1,156 cm-1 and 1,520 cm-1), protein (1,004 cm-1), fatty acids (1,444 cm-1 and 1,655 cm-1), and cytochrome (1,585 cm-1). The partial least squares discriminant analysis (PLS-DA) model achieved sensitivity and specificity rates of over 73%, although specificity was lower for gliosarcoma, at 50% [45].

Li Q et al. (2023) demonstrated in their studies that Raman spectroscopy offers distinct advantages for distinguishing glioma from normal brain tissue, including speed, non-invasiveness, and non-toxicity. Their approach utilized a peak detection method to automatically identify nine characteristic variables of Raman peaks, including peak position, intensity, and half-width. They analyzed 311 Raman spectra from 228 native tissue samples collected from 196 patients using a handheld Raman spectrometer optimized for rapid intraoperative glioma detection. Results showed a sensitivity of 87.21%, specificity of 86.49%, positive predictive value of 93.75%, negative predictive value of 74.42%, and overall accuracy of 86.99% [46].

Zhang L et al. (2023) presented findings based on 2,220 visible resonance Raman spectroscopy (VRRS) spectra from 63 unprocessed glioma samples using a VRR-LRR^TM^ Raman analyzer. Differences between glioma and normal brain tissue were observed in both the “fingerprint” region and the high wavenumber region, highlighting distinct molecular signatures, such as carotenoids, proteins, and lipids, between glioma and healthy tissues of different grades. The accuracy of distinguishing gliomas from normal tissue was above 80% when compared to standard histopathological results. This VRR-LRR^TM ^Raman analyzer holds potential as a new, label-free tool for real-time optical molecular pathology, enabling intraoperative glioma detection and aiding in tumor boundary delineation to maximize safe resection and preserve surrounding healthy tissue [47].

In a 2021 study, Hollon T analyzed three Raman-based imaging techniques in neuro-oncology: Raman spectroscopy (RS), coherent anti-Stokes Raman scattering (CARS) microscopy, and stimulated Raman histology (SRH). RS enables chemical characterization of tissue, distinguishing both normal and tumor-infiltrated tissues based on variations in macromolecular content in both ex vivo and in vivo settings. Coherent Raman imaging techniques, such as CARS and SRH, achieved sub-micron resolution, allowing for the detection of microscopic tumor infiltration in native brain tumor samples [48].

Pekmezci M et al. (2021) applied SRH to identify residual tumor presence in 82 out of 167 native samples obtained from tumor border areas. This is compared to residual tumor confirmation via immunohistochemistry (ICH) in 72 of 128 samples (56%) and hematoxylin-eosin staining in 82 of 169 samples (49%) [49].

Li JF et al. (2010) were the first to report the use of metal-based substrates in RS to improve brain glioma diagnosis. They demonstrated that silver (Ag), gold (Au), and copper (Cu) substrates, especially with rough surfaces or in nanoparticle (NP) form, are essential to achieve the surface-enhanced Raman scattering (SERS) effects necessary for sensitive detection-though this presents practical limitations for widespread use. Their work introduced an approach called shell-isolated nanoparticle-enhanced RS, where a monolayer of nanoparticles, or “smart dust,” is applied over the probed surface to amplify the signal [50].

Recent studies by McCabe SM et al. (2023) and Kenry et al. (2022) further emphasize that SERS imaging with nanoparticles provides remarkable sensitivity, serving as a foundation for molecular targeting technologies that enable multiplex and multimodal imaging. The development and optimization of contrast agents have been crucial for clinical integration in oncology [51,52]. Additionally, researchers have used RS to study aberrant glycosylation in blood plasma proteins as a marker for glioma progression [53].

Another research team, including Vrazhnov D et al. (2023), utilized spectroscopy on murine serum samples derived from mice implanted with U87 human glioblastoma cells to investigate serum markers for glioblastoma diagnosis [54]. This team is also exploring the application of terahertz spectroscopy combined with machine learning for early detection of human brain gliomas [55].

Bukva M et al. (2021) examined the potential of Raman spectroscopy for diagnosing CNS tumors through blood serum analysis. They collected 138 serum samples across four patient groups: glioblastoma multiforme, brain metastases from non-small cell lung cancer, meningioma, and lumbar intervertebral disc herniation (control group). Their findings demonstrated significant group differences, with diagnostic accuracy ranging from 82.9% to 92.5%, sensitivity between 80% and 95%, and specificity from 80% to 90%. The area under the curve (AUC) values ranged from 0.82 to 0.9, indicating high classification performance. These results suggest that Raman spectroscopy of blood serum, particularly in isolating small noncellular vesicles, holds significant potential for CNS tumor diagnostics [56].

Intraoperative diagnosis of normal brain tissue and gliomas

In 2013, Ji M et al. pioneered the use of stimulated Raman scattering (SRS) microscopy to differentiate between healthy and tumor brain tissue in human and murine models based on histoarchitectural and biochemical variations. SRS microscopy enabled the distinction between tumor and normal tissue in a murine xenograft model of human glioblastoma by identifying unique Raman spectra for each. The study also demonstrated a high correlation between SRS microscopy and traditional hematoxylin and eosin staining for detecting glioma infiltration (κ = 0.98). Additionally, SRS microscopy identified tumor boundaries in vivo during surgery on mice that were undetectable using standard techniques, suggesting that SRS may enhance both the safety and precision of surgical resection for diffuse gliomas through rapid intraoperative brain tissue assessment [57].

In 2017, Spencer L and Daniel O highlighted the potential of SRS microscopy for achieving high-accuracy tumor detection in brain tissue imaging. However, clinical implementation faced challenges due to the need for a switchable laser system with a reliable ultrafast dual-wavelength source [58].

Jin Z et al. (2022) identified extracellular acidosis caused by tumor cells as a dependable marker for detecting infiltrative tumor sites. They reported a SERS navigation system capable of delineating glioma boundaries without introducing exogenous probes. This approach significantly improved post-surgery survival in animal models compared to conventional clinical methods, indicating strong potential for clinical application in the resection of infiltrative tumors [59].

In 2023, Zhang Y demonstrated the high potential of Raman spectroscopy as a non-invasive and precise diagnostic tool for brain gliomas. However, challenges persist in distinguishing glioma patterns from normal brain tissue due to spectral artifacts caused by operator error or environmental changes. To address these issues, Zhang et al. proposed an outlier detection algorithm to increase model reliability and generalizability by filtering out anomalous data points [60].

The use of RS for rapid intraoperative tissue identification and glioma boundary determination has been documented in cases where tissue labeling with 5-aminolevulinic acid (5-ALA) is not feasible [61,62]. Jermyn and colleagues adapted RS for the operating room by developing a portable, contact-based fiber-optic probe that selectively measures Raman signals from brain tissue, isolating them from background noise. They tested the probe on brain tissue from 17 patients with grade 2-4 gliomas, comparing results against 161 biopsy samples, and achieved an accuracy of 92% in distinguishing invasive and dense glioma cells [63].

In a subsequent study, Jermyn’s team applied a nonparametric artificial neural network (ANN) model to filter out light artifacts in Raman spectra, enhancing the precision of tumor-brain tissue differentiation in vivo. This adjustment improved the method’s reliability for brain tumor detection, facilitating its integration into neurosurgical procedures. Post-filtration, the diagnostic accuracy of both RS techniques was ≥ 89% [64].

Desroches and colleagues analyzed high-wavenumber RS using a modified portable contact probe in 19 adult patients undergoing open brain surgery. During each procedure, a sterile probe was applied to brain tissue in the resection area, capturing RS spectra. To minimize ambient light interference, the neurosurgical microscope light was briefly turned off for each measurement. Following RS acquisition, the probe was rotated 180 degrees to collect a small tissue sample from the examined area, which was later fixed, paraffin-embedded, stained with hematoxylin-eosin, and examined histologically. Using a double-validation method, the authors reported that high-wavenumber RS could detect solid tumors with over 60% cancer cell content in situ, achieving 80% sensitivity and 90% specificity during surgery [65].

In 2019 and 2020, Bikmukhametova LR et al. also demonstrated RS’s capability for tumor diagnosis and intraoperative demarcation. Their findings highlighted distinct spectral differences associated with lipids, proteins, and nucleic acids, supporting RS’s potential as an optical biopsy tool for brain tumors. The study further emphasized the development of a comprehensive reference database of spectral components found in glial tumors, enabling multidimensional diagnostics and tumor boundary identification during surgery [6,11].

In 2022, Jabarkheel R et al. explored RS for precise intraoperative brain tumor diagnosis in pediatric patients. Using a rapid RS acquisition device, they visualized unprocessed, small ex vivo brain tissue samples from 29 pediatric patients. These samples underwent histopathological examination, and a dataset of 678 unique spectra from 160 samples was compiled. This dataset was subsequently used to develop a machine-learning model to distinguish between normal and tumor tissue as well as between normal and low-grade glioma tissue [66].

In 2023, Li Q et al. analyzed 769 Raman spectra from gliomas and 136 from normal brain tissue, corresponding to 205 and 37 cases, respectively. To increase the dataset for normal tissue spectra, they proposed a data augmentation algorithm using Gaussian kernel density estimation, expanding the normal tissue spectra to 600. This algorithm, which introduced a weighting factor based on Gaussian density, enhanced sample diversity and model reliability, achieving 91.67% accuracy, sensitivity, and specificity [67].

Riva M and colleagues published RS findings in 2021 based on native brain biopsy specimens, aiming to identify novel Raman bands to distinguish glioma from normal tissue. They analyzed 63 biopsy samples within minutes of sampling, collecting 3,450 spectra, with 1,377 classified as healthy tissue and 2,073 as tumor tissue. This approach achieved an 83% accuracy for distinguishing tumors from healthy tissue and identified 19 new Raman shifts with biological relevance, supporting RS as an effective ex vivo tool for isolating glioma tissue. This study contributes valuable spectroscopic data, advancing RS as a potential intraoperative tool for glioma detection [68].

In a 2022 trial, Herta J et al. compared the effectiveness of 5-ALA and RS in identifying tumor-infiltrated tissue in glioblastoma patients. In peritumoral regions, RS showed higher sensitivity than 5-ALA for tumor cell detection (69% vs. 46%) but lower specificity (57% vs. 81%). Combining RS with 5-ALA increased detection accuracy by approximately 10%. With further advancements in RS technology and integration with protoporphyrin IX fluorescence, this combination may enable more complete tumor resections in the future [69].

Intraoperative diagnosis of tumor boundaries

Research teams led by Zhang L et al. and Zhang Z et al. (2023) conducted studies on the use of Raman spectroscopy (RS) for the intraoperative diagnosis of gliomas, differentiation of normal brain tissue, and identification of tumor boundaries during resection. Their findings suggest that RS is a highly promising tool for diagnosing brain gliomas due to its non-invasive nature and high data density [47,70].

In related research from 2018 and 2019, Zhou Y. explored RS for verifying glioma boundaries during surgical removal. In one study, Zhou Y et al. (2018) presented results using the VRRS method to differentiate gliomas and delineate tumor boundaries. They detected 87 VRRS spectra across 21 human brain samples from 4 tissue types, including control and glioma tissues of grades II, III, and IV. This analysis highlighted the discovery of two new Raman peaks at 1,129 cm⁻¹ and 1,338 cm⁻¹, associated with vibrational couplings in brain tissues. These peaks, showing enhanced resonance, correlate with increased levels of lactic acid/phosphatidic acid and adenosine 5′-triphosphate (ATP)/nicotinamide adenine dinucleotide (NAD), respectively. Findings indicated that concentrations of lactic acid and ATP vary with glioma grade, with higher malignancy associated with increased levels of these metabolites [71].

In another 2019 paper, Zhou and colleagues presented a VRRS-based approach for determining glioma boundaries and grading. This method identifies specific diagnostic spectral biomarkers based on tissue composition changes, including molecular vibrational fingerprints of carotenoids, tryptophan, amides I/II/III, proteins, and lipids. These biomarkers are used to distinguish glioma tissue from normal brain tissue and to characterize glioma properties. Cross-validation yielded sensitivity, specificity, and accuracy rates of 100%, 96.3%, and 99.6%, respectively, in distinguishing glioma from normal brain tissue. The accuracy for distinguishing low-grade (I and II) from high-grade (III and IV) gliomas was 96.3%, 53.7%, and 84.1%, respectively, with an overall accuracy of 75.1% [72].

Using SERS, Yang G and colleagues identified a pH decrease in tumor tissue compared to healthy brain tissue, providing a refined means of delineating the precise boundary between tumor and healthy brain tissue [73].

Liu J and colleagues (2024) submit typical spectra of biological samples that require large-area imaging spectra from multiple locations. In vivo detection may further extend procedure times, potentially compromising patient safety. The authors argue that it must comply with the balance between acquisition time and spectrum quality [74].

**Figure 2:**
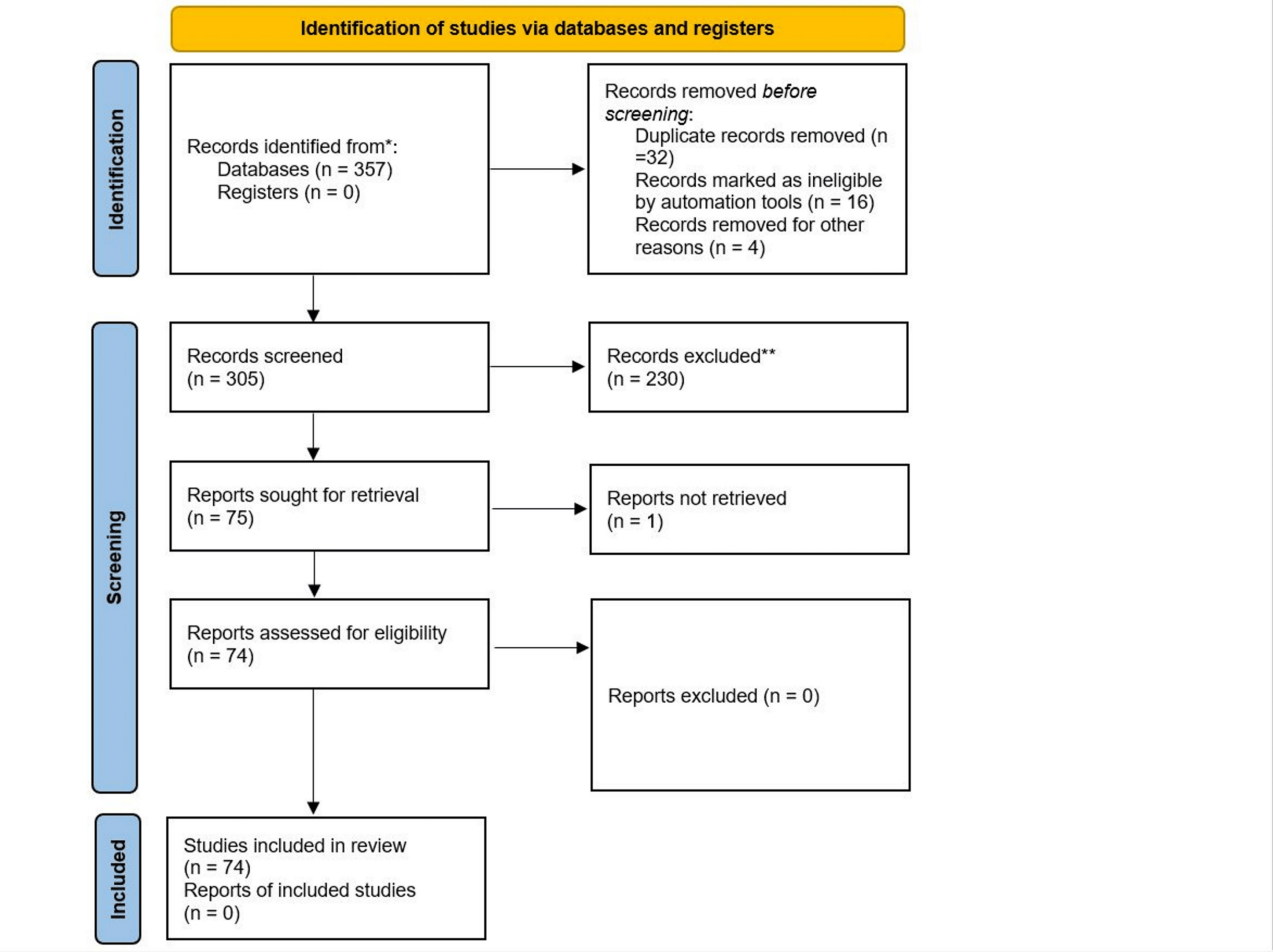
Identification of studies via databases and registers

RS and other forms of vibrational spectroscopy have shown considerable promise in neurosurgery for enhancing the identification and management of both normal and abnormal brain tissues [27]. These techniques enable precise differentiation between healthy tissue and gliomas, including intraoperative delineation of tumor boundaries, and facilitate rapid, non-destructive testing of biopsy specimens, frozen tissue, and paraffin-embedded tissue blocks (Table 1). Recent advancements have also expanded their use to analyze body fluids, such as blood plasma, for tumor detection and monitoring postoperative tumor growth. The comprehensive study selection process for this review is illustrated in the PRISMA flow diagram (Figure 2) [75]. These studies collectively highlight RS's potential to revolutionize neuro-oncology by providing high-accuracy, real-time diagnostic and monitoring tools that address the limitations of current intraoperative imaging and molecular diagnostic techniques.

**Table 1:** Applications of different types of spectroscopy

Type of spectroscopy	Use cases	References
Surface-Enhanced Raman Spectroscopy (SERS)	Enhances the Raman signal using a metal-coated substrate. Useful for determining tumor boundaries by pH differences between tumor and healthy brain tissue. Limited intraoperative application.	[28-31,39,50,51,59,73]
Intraoperative Raman Probes	Allows rapid delineation of tumor borders from healthy tissue. Requires filtering of background signals and light artifacts using artificial intelligence.	[6,11,27,32,33,49,61-65,70-72]
Stimulated Raman Histology (SRH)	Applicable to both native and preserved tissues (frozen, formalin-fixed, paraffin-embedded). The use of 5-ASA enhances diagnostic accuracy.	[13,22,31,35-38,40-44,46,48,49]
Raman Spectroscopy for Blood Plasma Analysis	Potential for diagnosing CNS tumors and monitoring tumor progression postoperatively.	[53,54,56]

## Conclusions

RS has emerged as a transformative technology in neuro-oncology, leveraging the ability to identify molecular "fingerprints" unique to biological tissues and molecules through inelastic light scattering without tissue destruction. Among its advancements, SRH stands out for its broad applications and clinical benefits. SRH simplifies the analysis of native biopsy specimens, frozen tissues, paraffin-embedded tissue blocks, and blood plasma, offering rapid, non-destructive insights into tissue composition. Additionally, RS enables precise intraoperative differentiation between healthy brain tissue and various glial tumor types, enhancing surgical precision. It also aids in delineating glioma boundaries, a critical factor in achieving optimal tumor resection while preserving healthy tissue. By reducing diagnostic time, facilitating real-time surgical decision-making, and potentially shortening surgery duration, RS is poised to become an indispensable tool in neurosurgery and neuro-oncology, significantly improving the management and treatment of brain gliomas.
